# Combined skeletal dysplasia and vasculopathy phenotypes associated with in-frame intragenic deletion in *PRKACA*

**DOI:** 10.1016/j.xhgg.2026.100626

**Published:** 2026-05-18

**Authors:** K. Nicole Weaver, Jan W. Broeckel, Kari Brown, Halley Wasserman, Jian Wu, Ashley Neal, Carlos E. Prada, Neil Lennart Kleinschmidt, Erik M.F. Machal, Daniela Bertinetti, Victor L. Ruiz-Perez, Susan S. Taylor, Samantha A. Brugmann, Friedrich W. Herberg

**Affiliations:** 1 The Heart Institute, Division of Cardiology Cincinnati Children’s Hospital, Cincinnati, Ohio, USA; 2 Department of Pediatrics, University of Cincinnati College of Medicine, Cincinnati, Ohio, USA; 3 Division of Human Genetics, Cincinnati Children's Hospital, Cincinnati, OH 45229, USA; 4 Department of Biochemistry, University of Kassel, Kassel, Germany; 5 Division of Developmental Biology, Cincinnati Children’s Hospital, Cincinnati, OH, USA; 6 Division of Endocrinology, Cincinnati Children’s Hospital, Cincinnati, OH, USA; 7 Department of Pharmacology, University of California, San Diego, La Jolla, CA 92093, USA; 8 Edwards Family Division of Genetics and Rare Diseases, Lurie Children’s Hospital and Department of Pediatrics, Northwestern University Feinberg School of Medicine, Chicago, IL, USA; 9 Technical University Dresden, Center for Molecular and Cellular Bioengineering (CMCB), Dresden, Germany; 10 Instituto de Investigaciones Biomédicas Sols-Morreale, Consejo Superior de Investigaciones Científicas (CSIC)-Universidad Autónoma de Madrid, Madrid, Spain; 11 CIBER de Enfermedades Raras (CIBERER), Instituto de Salud Carlos III (ISCIII), Madrid, Spain; 12 Department of Biochemistry and Molecular Biophysics, University of California, San Diego, La Jolla, CA 92093, USA

**Keywords:** protein kinase a, skeletal dysplasia, vasculopathy, deletion

## Abstract

Pathogenic missense variants in *PRKACA* cause craniofacial, skeletal, and cardiac defects similar to Ellis-van Creveld syndrome. We report an individual with a previously unreported, *de novo* 3-amino-acid deletion in *PRKACA*, identified on trio genome sequencing, and phenotypic features including severe neonatal hypotonia, appendicular skeletal abnormalities, osteopenia, aortic dilation, coronary dilation, and vascular tortuosity. To assess this variant’s effects, we performed *in vitro* and *in vivo* studies, generated an *in silico* model, and assessed cell ciliation in induced pluripotent stem cells from the patient. Although the protein product of the PRKACA (Protein kinase A [PKA]-Cα) 3-amino-acid-deletion variant is catalytically active, it shows reduced interaction with the regulatory subunits of PKA (particularly type II), resulting in overactivation of the PKA pathway and/or an inability to initiate Hedgehog signaling. The deletion affects a key portion of PKA-C important for substrate tethering. Patient-derived induced pluripotent stem cells (iPSCs) have reduced ciliation compared to controls. Collectively, this supports that the *PRKACA* variant is pathogenic, and we propose that it is causal for our patient’s unique skeletal dysplasia and vasculopathy phenotypes. This expands the phenotypic spectrum of pathogenic variants in *PRKACA* and suggests that affected individuals may require periodic screening for aortic and coronary dilation as well as osteopenia.

## Teaser

Weaver et al. describe a patient with skeletal dysplasia and vasculopathy phenotype in association with a previously unreported intragenic deletion of *PRKACA*, and they present functional data supporting pathogenicity of the variant.

## Introduction

Protein kinase A (PKA) is ubiquitously expressed, and its kinase activity is tightly regulated. PKA forms an inactive holoenzyme with two catalytic (C) subunits and a regulatory (R) subunit dimer (R_2_C_2_).^1^^,^^2^^,^^3^ Activation and inhibition of PKA activity is primarily controlled via its R-subunits binding to cyclic AMP (cAMP). There are four functionally non-redundant R-subunits (RIα, RIβ, RIIα, and RIIβ) encoded by four genes (*PRKAR1A* [MIM: 188830], *PRKAR1B* [MIM: 176911], *PRKAR2A* [MIM: 176910], and *PRKAR2B* [MIM: 176912]). When cytosolic cAMP concentrations are high, the activity of the C-subunits, Cα and Cβ, encoded by the genes *PRKACA* and *PRKACB*, is unleashed.^3^ Conversely, when cAMP is low, the R-subunits bind and inhibit the C-subunits. Five previously reported pathogenic variants in *PRKACA/B* are associated with cardioacrofacial dysplasia type 1 (*PRKACA*) and type 2 (*PRKACB*) (MIM: 619142 and 619143).^4^ Individuals with these variants (one recurrent variant in *PRKACA*, NM_002730.4: c.409G>A [p.Gly137Arg], and four variants in *PRKACB*, NM_002731.3: c.703G>C [p.Gly235Arg], c.262C>A [p.His88Asn], c.263A>G [p.His88Arg], c.161C>T [p.Ser54Leu]) share the same phenotype, consistent with an Ellis-van Creveld syndrome type ciliopathy (atrioventricular septal defects, post-axial polydactyly, short limbs, and ectodermal abnormalities involving nails and teeth).^4^^,^^5^^,^^6^ Those variants facilitated dissociation of the C-subunits from the R-subunits at low cAMP levels and thus cause overactivation of PKA signaling. One of PKA’s targets is the transcription factor GLI3. When GLI3 is phosphorylated, it undergoes C-terminal processing and becomes a strong repressor of Hedgehog (Hh) signaling. Besides inhibition by the R-subunits (canonical pathway), inhibition of C-subunit activity is caused by the noncanonical interaction of PKA C-subunits with the C-terminal tail of the Hh component SMO.^7^ When specific *PRKACA/B* missense variants are present, the cell’s ability to respond to activation of Hh signaling is reduced because the corresponding C-subunits resulting from these variants remain in part dissociated from R-subunits and catalytically active despite low levels of cAMP.^4^ This is a similar effect to what occurs if *EVC2* or *EVC* (Ellis-van Creveld syndrome-associated genes) are deleted. After stimulation of the Hh pathway with Smoothened agonist (SAG), EVC or EVC2 knockout cells exhibit reduced expression of Hh-target genes and increased levels of GLI3-repressor compared to control cultures.^8^ Here, we describe a child with different phenotypes in association with a previously unreported, putative pathogenic variant in *PRKACA*, c.870_878del (p.Asn290_Ile292del). We biochemically characterize the mutation and correlate this site with the structure of PKA-Cα, collectively providing evidence in support of variant pathogenicity and expanding the phenotype and genotype spectrum of *PRKACA*-related cardioacrofacial dysplasia type 1.

## Material and methods

### Participant consent

Informed consent was obtained from the patient’s parents (IRB #2016-7017, principal investigator K.N.W.) to obtain a blood sample for induced pluripotent stem cell creation, and for publication of this report.

### BRET assays

Bioluminescence resonance energy transfer (BRET) assay and protein modeling were performed as described in Palencia-Campos et al.^4^ Briefly, cultured HEK293 cells were seeded in a white 96-well microplate (Nunclon Delta Surface; Thermo Scientific) with a density of 2 × 10^4^ cells per well in Dulbecco’s modified Eagle’s medium with 10% fetal calf serum (Capricorn Scientific). The cells were transiently transfected with GFP^2^-tagged PKA-Cα wild type (WT) or the *PRKACA* patient deletion (del NDI) and the respective Rluc8-tagged PKA regulatory subunit (RIα or RIIα) using polyethyleneimine (25 kDa, Polysciences).^9^ The reporter proteins were expressed for 48 h at 37°C and 6% CO_2_. Transfected cells were washed with Hanks’ balanced salt solution (HBSS; Biowest) before starting the reaction with the addition of 5 μM coelenterazine 400A with a total measuring time of 20 min. After 5 min, the increase of intracellular cAMP levels was stimulated by injections of either isoproterenol (Sigma-Aldrich) or a mixture of 50 μM forskolin (Sigma-Aldrich) and 100 μM 3-isobutyl-3-methylxanthine (IBMX) (Sigma-Aldrich) in HBSS containing 5 μM coelenterazine 400A. BRET^2^ ratios were measured using a POLARstar microplate reader (BMG Labtech) and calculated from the 515 nm (GFP^2^ signal) to 410 nm (luciferase signal) ratio, and the zero signal was normalized against an Rluc control vector. Data were evaluated with GraphPad Prism 10.0 by plotting the normalized BRET^2^-ratio against time.

### Induced pluripotent stem cell studies

Patient and control peripheral blood mononuclear cells (PBMCs) and induced pluripotent stem cells (iPSCs) used in this study were generated by the Cincinnati Children’s Pluripotent Stem Cell Facility. PBMCs were isolated from blood samples using Lymphoprep and SepMate tubes (StemCell Technologies) and cryopreserved in 90% defined fetal bovine serum (FBS) (Hyclone)/10% DMSO. For reprogramming, PBMCs were thawed in erythroid expansion medium (EEM) consisting of StemSpan SFEM II containing 1× StemSpan Erythroid Expansion Supplement (StemCell Technologies) and cultured for 8 days. After PBMC priming (designated as d0), cells were transduced in EEM using Sendai viral vectors co-expressing reprogramming factors Oct4, Klf4, Sox2, and cMyc (Cytotune 2.0, Thermo Fisher Scientific). On day 3, transduced cells were then plated on Cultrex (BioTechne)-coated in 2 mL of EEM. On day 5, 1 mL of fresh ReproTeSR (StemCell Technologies) medium was added to the wells. On day 6, 1 mL of fresh ReproTeSR was added to the existing medium in each well. Starting on day 7, wells underwent a complete daily medium change with 2 mL of ReproTeSR medium. Putative iPSC colonies were then manually excised and replated in feeder-free culture conditions consisting of stem cell qualified Cultrex (BioTechne) and mTeSR1 (StemCell Technologies). Lines exhibiting robust proliferation and maintenance of stereotypical human pluripotent stem cell morphology were then expanded and cryopreserved at ∼passage 10. iPSC colonies were maintained in mTesR1 medium (StemCell Technologies) and cultured on Cultrex SCQ-coated plates (BioTechne, 3434-010-02). Cells were passaged once a week using GCDR (StemCell Technologies) and plated on Cultrex SCQ-coated coverslips for experiments. iPSCs were differentiated into neural crest cells (NCCs) with the STEMDiff Neural Crest Differentiation Kit (StemCell Technologies) according to the company instructions. Briefly, one well of iPSCs was detached with Accutase (Thermo Fisher) into single cells. Cells were resuspended in provided medium containing 10 μM Y-27632 (Cell Signaling, 13624S) and plated at 8.6 × 10^4^ cells/cm^2^ on Cultrex SCQ-coated 12-well plates. Cells were cultured with daily medium changes without Y-27632. On day 6, cells were passaged with Accutase on Cultrex SCQ-coated coverslips for experiments.

Cells were fixed in 4% PFA for 15 min, washed three times with PBS, and blocked in 5% normal goat serum in PBS-T for 30 min. Primary antibody Arl13b (1:500, Proteintech, 17711-1-AP) was added in blocking buffer at 4°C overnight. Goat anti-rabbit Alexa 488 secondary antibody was used at 1:1,000 in blocking buffer for 1 h at room temperature. Coverslips were placed on slides with Prolong Gold plus DAPI for nuclear staining and imaged on Leica DM5000B.

## Results

### Proband phenotype and clinical testing

**Figure 1:**
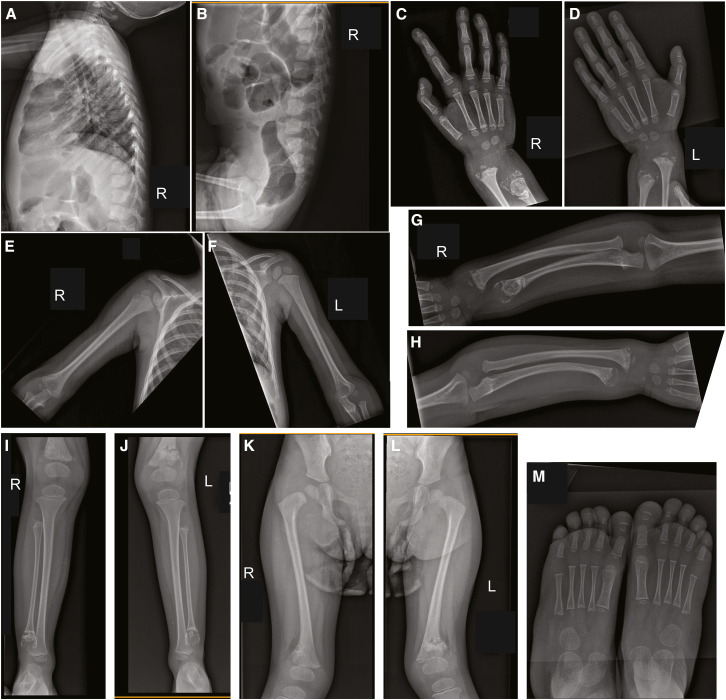
Skeletal survey of individual with *PRKACA* deletion at 27 months of age with multiple anomalies (A and B) Short ribs with anterior scalloping. (C and D) Increased number of metacarpal pseudo-epiphyses, two ossification centers in thumb distal tufts, and hypoplastic tufts of digits 2–5. (E and F) Normal humeri. (G and H) Severely hypoplastic/short ulna and distal radius is small and fragmented. Distal radial and ulnar metaphyseal fragmentation. Distal radial epiphyses are small and fragmented. (I and J) Severely hypoplastic/short fibula bilaterally with metaphyseal fragmentation at proximal and distal fibula. (K and L) Flat acetabular roof and widened sacro-sciatic notches. Distal femoral metaphyseal fragmentation. (M) Normal feet.

The proband was born at 36.8 weeks to a 36-year-old mother and 38-year-old father. Pregnancy was complicated by polyhydramnios and placental rupture. Birth weight was 3.289 kg (41^st^ percentile) and birth length was 51.4 cm (79^th^ percentile). She was admitted to the neonatal intensive care unit for 2 weeks for management of respiratory distress and hypotonia. Her initial echocardiogram demonstrated a large patent ductus arteriosus, which resolved by 2 months of age, and patent foramen ovale. Creatine phosphokinase level was normal. She required readmission at 2 months old for poor feeding and failure to thrive requiring placement of a gastrostomy tube. She was also diagnosed with obstructive sleep apnea, which required bi-level positive airway pressure. At 9 months old, she developed obstructive nephrolithiasis causing hydroureteronephrosis and urinary tract infection, necessitating stent placement. She continued to make developmental progress with the assistance of therapies and gained weight via gastrostomy-tube feeding. At age 20 months, echocardiogram demonstrated mild aortic root (1.9 cm, Pediatric Heart Network *Z* score +3.8) and mild ascending aorta dilation (1.5cm, *Z* score +2.1) with further progression in aortic root dilation by age 2 (2.3 cm, *Z* score +5.5). At that time, MRI angiography also identified tortuous intracranial vessels. Treatment with losartan was initiated. In addition to progressive aortic dilation, echocardiogram at age 5 years demonstrated diffuse dilation of the left main, left anterior descending, and circumflex coronary artery. Computed tomography scan of her heart excluded a coronary artery fistula. Thus, she was also started on aspirin for antiplatelet therapy. There was no known history of Kawasaki disease or other infection. Given concern for structural bone anomalies, a skeletal survey identified multiple abnormalities (Figure 1). Dual X-ray absorptiometry revealed low bone density for age at the lumbar spine (*Z* score −3.3 at 2.8 years of age, −3.1 at 4.4 years, −3.9 at 5.75 years), total body less head (*Z* score −4.2 at 4.4 years), and total hip (−6.2 at 5.75 years). Bone turnover markers (osteocalcin, C-terminal telopeptide) were normal for age. At age 5 years, she had surgery to address bilateral genu valgum, patellar dislocation, and distal femoral cartilaginous bar.

Amniocentesis was performed prenatally, and microarray was normal. Postnatally, a neuromuscular gene panel identified a single variant of uncertain significance in *GAA*. Subsequent enzyme testing for Pompe disease was normal. Clinical testing for myotonic dystrophy (*DMPK* CTG repeat analysis by PCR, long-read sequencing, Athena Diagnostics) and Prader Willi/Angelman syndrome (DNA methylation analysis, Quest Diagnostics) was also negative. Mitochondrial whole-genome sequencing and deletion/duplication testing (CCHMC [Cincinnati Children's Hospital Medical Center]) was normal. Clinical trio genome sequencing (Rady Children’s, San Diego) was sent, and no additional variants were reported. Clinical muscle biopsy was performed at 5 months of age. Electron microscopy was notable for several fibers with subsarcolemmal and/or perinuclear mitochondrial aggregates, although mitochondria had normal internal architecture and no inclusions; there were occasional atrophic fibers. Immunohistochemistry for alpha dystroglycan, collagen VI, and merosin were all comparable to controls. Nonspecific mild fiber size variation was noted. At age 2 years, clinical genome reanalysis was requested with new phenotype information provided, and a *de novo* deletion in *PRKACA* c.870_878del; p. (Asn290_Ile292del), classified as a variant of uncertain significance, was reported. Two variants of uncertain significance in *POMT1* were reported (c.687G>C [p. Gln229His], *de novo* but thought to be on paternal allele; and c.-83C>T, maternal). Given normal dystroglycan staining on muscle biopsy and lack of concordance between proband presentation and *POMT1*-associated phenotypes, further investigation of these variants was not pursued.

### Research analyses

**Figure 2:**
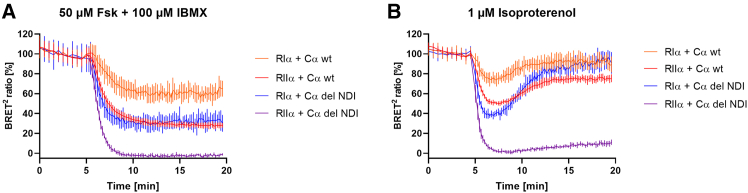
Holoenzyme dissociation measured using PKA BRET sensors PKA BRET sensor encompassing wild-type (WT) or Asn290_Ile292del (del NDI) PKA-C-subunits with either RIα or RIIα subunits were transfected into HEK293 cells and an increase in intracellular cAMP was stimulated using 50 μM forskolin/100 μM IBMX (A) or 1 μM isoproterenol as indicated on the plots (B). The decreases in BRET signals show the dissociation of the respective PKA holoenzymes. The PKA-Cα del NDI variant forms less-stable holoenzymes with either RIα or RIIα. Stimulation with isoproterenol (B) shows reassociation of PKA-Cα del NDI only for RIα but not RIIα. Data is presented as the mean (± SD) of six replicates from one transfection. Three independent experiments were performed for each condition (*n* = 3).

Given high suspicion that the *PRKACA* variant is the etiology for the patient’s phenotype, we performed *in vitro* biochemical assays to determine the effect of the deletion on PKA function.^7^ Recombinantly expressed Asn290_Ile292del PKA protein showed a lower specific kinase activity (9.0 ± 1.2 U/mg; *n* = 4) compared to WT protein (17.2 ± 1.2 U/mg; *n* = 3) based on a spectrophotometric kinase activity assay using the peptide substrate Kemptide.^10^ PKA BRET sensors encompassing WT and *PRKACA* variant were developed to follow holoenzyme dissociation after application of cAMP-stimulating drugs in HEK293 cells (Figure 2). *PRKACA* c.870_878del PKA holoenzymes formed with both RIα and RIIα regulatory subunit isoforms dissociated to a higher degree than their WT counterparts at full cAMP stimulation (forskolin/IBMX. Figure 2A). Importantly, the effect is more pronounced in RIIα holoenzyme, which dissociates completely even after stimulation with isoproterenol and does not show a fast reassociation after cAMP decreases due to the action of intracellular phosphodiesterases (Figure 2B).

**Figure 3:**
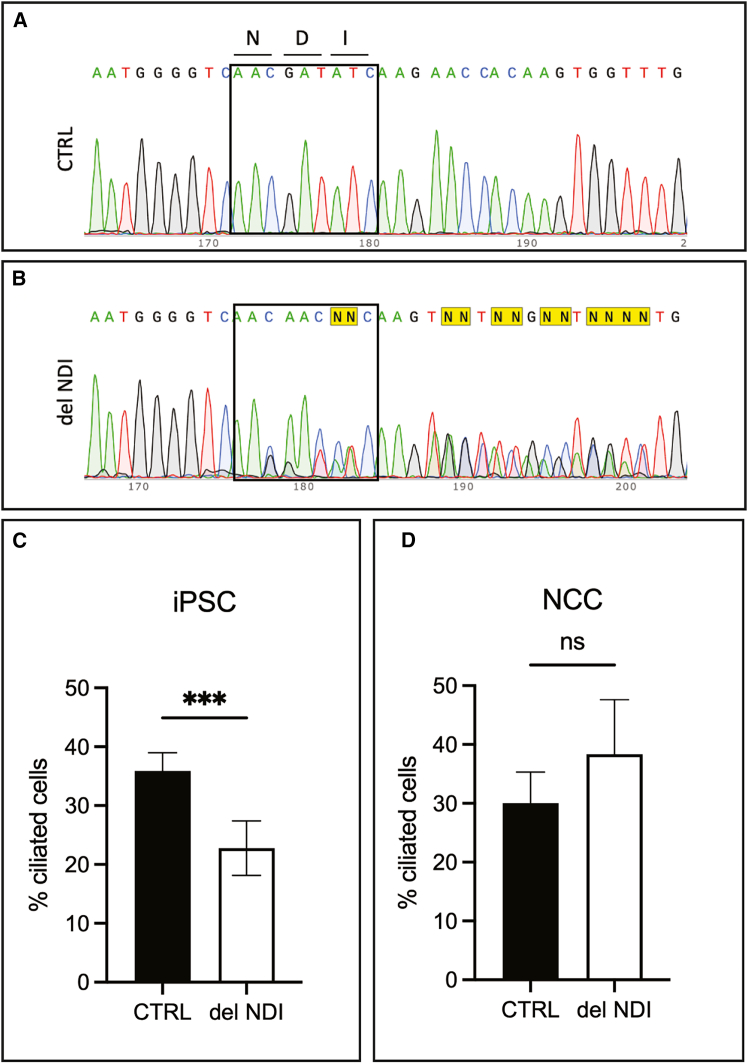
Patient derived undifferentiated iPSC have reduced ciliation while induced neural crest cells do not (A and B) Sanger sequencing confirms normal *PRKACA* sequence in control (A) and 9-bp deletion in patient iPSC (B). (C and D) Cilia counts on control and patient iPSCs (C) and neural crest cells (NCCs) (D); *n* = 4; 1,500 cells counted for each condition and analyzed by two-tailed unpaired *t* test (∗∗∗*p* < 0.0001; ns, not significant). Ciliated cell number is reduced in patient vs. control iPSC, but there is not a statistically significant difference in cell ciliation in patient vs. control iPSC-derived NCCs.

We also wanted to determine whether the *PRKACA* deletion might affect ciliation, given the known important role of PKA in Hh signaling and classification of Ellis-van Creveld syndrome as a ciliopathy. Patient and control iPSCs were stained with anti-ARL13B to mark the ciliary axoneme. This identified a significant reduction in ciliated cells in patient versus control iPSCs (22.78% vs. 38.10%, *p* = 0.0018) (Figure 3).

## Discussion

**Figure 4:**
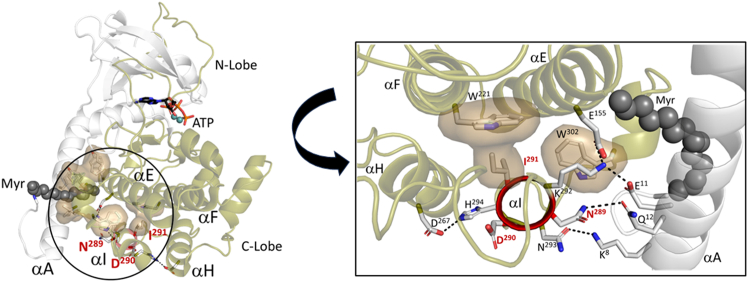
The residues N^289^DI^291^ in the αI helix are important in the PKA-C-subunit structure (Left) The crystal structure of myristylated PKA-C (PDB: 1CMK). The N and C lobes of PKA-C are colored in white and tan, respectively. The black circle highlights the key interactions of the residues N^289^DI^291^ (in red) and the position of the αI helix. The hydrophobic binding pockets of the myristoyl group, in black, are also shown as shells colored in sand. (Right) Close-up view of the interactions of the residues N^289^DI^291^ and αI helix. N^289^ (in red) together with αI helix residues K^292^, N^293^, and H^294^ form multiple hydrogen bonds with the residues from αA, αE, and αH helices. I^291^ hydrophobically packs to W^221^ from the αF helix.

We described an individual with a unique phenotype, overlapping yet distinct from Ellis-van Creveld syndrome, which we propose is due to a so-far undescribed *de novo* variant that results in a 3-amino-acid deletion of *PRKACA*. The deletion affects the I helix of the C-lobe of PKA-Cα (Figure 4), which is a central cog linking four helices as well as the myristylation site. It was identified previously as a tethering site for substrate proteins and is likely a key allosteric site.^11^^,^^12^ Interestingly, the phenotype of this patient exhibits striking similarities to a patient reported by Espiard et al.^13^ who has a single-amino-acid deletion in *PRKACB* (c.858_860GAA [p.K286del]) encoding PKA-Cβ. This previously reported individual has short stature, hypercalcemia with mild calciuria, unilateral renal cysts, renal calculus, metaphyseal irregularities and hypoplastic distal phalanges, joint dislocation, hypertelorism, and triangular facies. The *in vivo* BRET assays indicate that our patient’s deletion variant causes more severe overactivation of PKA signaling compared to the recurrent missense variant in *PRKACA* previously reported in the five individuals described above. Consistent with the previously hypothesized effect of *PRKACA* pathogenic variants on ciliary signaling,^4^ we found that our patient’s iPSCs have reduced ciliation. Further, we do not see the same phenotype in iPSC-derived NCCs, which suggests there may be tissue-specific effects of *PRKACA* alterations. While our functional studies of this variant are not sufficient to meet the American College of Medical Genetics^14^ PS3 criterion, we interpret the variant as likely pathogenic, meeting PS2 (*de novo*), PM2 (absence from controls), and PM4 (change in protein length) criteria.

Skeletal anomalies (short ribs, polydactyly, metaphyseal dysplasia) are strongly associated with ciliopathy syndromes, and cilia play an important role in skeletal development.^15^ Abnormalities of bone density have rarely been described in ciliopathy patients, but recent reports document abnormal bone density in *PRKACA/B* patients (P7 with osteoporosis in Palencia-Campos et al.,^4^ P1 with osteopenia in Sithambaram et al.^6^). The individual we describe has consistently low bone mineral density assessed by DEXA scan but has not yet had any confirmed fragility fractures. Treatment with bisphosphonate infusions has been considered to address the low bone density but was not initiated given lack of confirmed fragility fractures. Regarding other endocrinological phenotypes, previous studies found adrenal disorders in individuals with germline gene duplications of *PRKACA*,^16^ which are not present in our patient. This is in good agreement with findings that missense variants in *PRKACA* lead to developmental phenotypes lacking adrenal disorders.^4^

Aortic dilation, coronary dilation, and vascular tortuosity have not been reported thus far in association with ciliopathies. Interestingly, PKA is expressed in endothelial cells, and PKA inhibition can result in excessive angiogenesis.^17^ Autosomal-dominant polycystic kidney disease, also a ciliopathy, is associated with vasculopathy features including aortic dilation, intracerebral aneurysm predisposition, and coronary artery dissection.^18^ While the possibility of unrecognized or incomplete Kawasaki disease cannot be entirely excluded, aortic dilation is not a common presentation of Kawasaki disease and, when present, is not expected to be progressive.^19^^,^^20^ Collectively this, plus lack of any other identified vasculopathy/aortopathy variants in her genome, provides circumstantial evidence that the proband’s vasculopathy phenotype may also be secondary to her *PRKACA* deletion, although further studies are needed to confirm this association. Given that the vascular phenotype present in our patient has not previously been reported with this genetic variant, the benefit of medical therapy to reduce risk of dissection or slow the rate of aortic dilation is unclear. However, she was started on an angiotensin-receptor blocker, consistent with recommendations from the recent pediatric aortopathy scientific statement.^21^ Given the degree of her coronary dilation, aspirin was initiated extrapolating from the Kawasaki disease literature supporting antiplatelet therapy for children with persistent coronary aneurysms.^22^

In summary, we present a compelling case for expanding the genotype and phenotype spectrum of *PRKACA*-related disease, presenting evidence of pathogenicity for an intragenic deletion in our patient as well as evidence for previously unappreciated involvement of vasculature and bone mineralization.

## Data code and availability

Additional clinical information will not be made available due to patient privacy protection.

## Acknowledgments

The authors wish to thank the Pluripotent Stem Cell Facility at CCHMC for generation of iPSC from patient PBMCs. We also thank the patient and their family for their generosity in allowing us to share this story, and Dr. Ralph Lachman for his expertise in reviewing and interpreting skeletal X-rays. S.S.T. acknowledges financial support from the 10.13039/100000002National Institutes of Health GM130389. F.W.H. and J.W.B. acknowledge financial support from the 10.13039/501100001659German Research Foundation (DFG)-funded Research Training Group 448909517/GRK 2749: Biological Clocks on Multiple Time Scales.

## Declaration of interests

The authors declare no competing interests.
